# Estimating HIV-1 Genetic Diversity in Brazil Through Next-Generation Sequencing

**DOI:** 10.3389/fmicb.2019.00749

**Published:** 2019-04-09

**Authors:** Brunna M. Alves, Juliana D. Siqueira, Isabel M. Prellwitz, Ornella M. Botelho, Vanusa P. Da Hora, Sabri Sanabani, Patrícia Recordon-Pinson, Hervé Fleury, Esmeralda A. Soares, Marcelo A. Soares

**Affiliations:** ^1^Programa de Oncovirologia, Instituto Nacional de Câncer, Rio de Janeiro, Brazil; ^2^Laboratório de Biologia Molecular, Escola de Medicina, Universidade Federal do Rio Grande, Rio Grande do Sul, Brazil; ^3^LIM-3, Hospital das Clinicas FMUSP, Faculty of Medicine, University of São Paulo, São Paulo, Brazil; ^4^CNRS MFP-UMR 5234, University Hospital of Bordeaux, University of Bordeaux, Bordeaux, France; ^5^Departamento de Genética, Universidade Federal do Rio de Janeiro, Rio de Janeiro, Brazil

**Keywords:** HIV-1, genetic diversity, NGS, NFLG, subtype

## Abstract

Approximately 36.7 million people were living with the human immunodeficiency virus (HIV) at the end of 2016 according to UNAIDS, representing a global prevalence rate of 0.8%. In Brazil, an HIV prevalence of 0.24% has been estimated, which represents approximately 830,000 individuals living with the virus. As a touristic and commercial hub in Latin America, Brazil harbors an elevated HIV genetic variability, further contributed by the selective pressure exerted by the host immune system and by antiretroviral treatment. Through the progress of the next-generation sequencing (NGS) techniques, it has been possible to expand the study of HIV genetic diversity, evolutionary, and epidemic processes, allowing the generation of HIV complete or near full-length genomes (NFLG) and improving the characterization of intra- and interhost diversity of viral populations. Greater sensitivity in the detection of viral recombinant forms represents one of the major improvements associated with this development. It is possible to identify unique or circulating recombinant forms using the near full-length viral genomes with increasing accuracy. It also permits the characterization of multiple viral infections within individual hosts. Previous Brazilian studies using NGS to analyze HIV diversity were able to identify several distinct unique and circulating recombinant forms and evidenced dual infections. These data unveiled unprecedented high rates of viral recombination and highlighted that novel recombinants are continually arising in the Brazilian epidemic. In the pooled analysis depicted in this report, HIV subtypes have been determined from HIV-positive patients in five states of Brazil with some of the highest HIV prevalence, three in the Southeast (Rio de Janeiro, São Paulo, and Minas Gerais), one in the Northeast (Pernambuco) and one in the South (Rio Grande do Sul). Combined data analysis showed a significant prevalence of recombinant forms (29%; 101/350), and a similar 26% when only NFLGs were considered. Moreover, the analysis was able to evidence the occurrence of multiple infections in some individuals. Our data highlight the great HIV genetic diversity found in Brazil and unveils a more accurate scenario of the HIV evolutionary dynamics in the region.

## Introduction

The first registry of AIDS was reported in the beginning of the 1980s, and until now 77 million people have become infected with human immunodeficiency virus (HIV) and 35 million have died from AIDS-related causes. By 2017, it was estimated that 36.9 million people worldwide were living with HIV (UNAIDS, 2018). Harboring over one-third of the total population of Latin America, Brazil accounts for nearly half of the new HIV infections and of the estimated total of individuals living with HIV/AIDS (48 and 46%, respectively) in the region (UNAIDS, 2018). This scenario, along with the high error-prone rate of the viral reverse transcriptase (RT), high virus replication rates and recombination events, contributes to the remarkable accumulation of genetic diversity in its population during the course of infection, further influenced by selective pressure exerted by the host immune system and by antiretroviral treatment (Roberts et al., 1988; Overbaugh and Bangham, 2001; Zhuang et al., 2002; Santoro and Perno, 2013).

The surveillance of HIV diversity assists to monitor the emergence of new subtypes and the presence of novel strains in a given geographic location (Hemelaar, 2013). The great diversity of HIV-1 group M, which disseminated on a global scale and dominates the current AIDS pandemic, allowed the phylogenetic classification in nine pure subtypes (A–D, F–H, J, and K), sub-subtypes (A1–A5, F1–F2), circulating recombinant forms (CRFs) and unique recombinant forms (URFs) (Robertson et al., 2000). Currently, more than 90 CRFs have been reported in the HIV Sequence Database of the United States Los Alamos National Laboratory^1^. Recombinant viruses are the result of simultaneous infection by multiple viruses during a single transmission event (co-infection) or from sequential infection at multiple transmission events (superinfection) (Yerly et al., 2004). Molecular epidemiology studies show that the overall distribution of HIV-1 groups, subtypes and recombinant forms is highly heterogeneous, with significant differences in the size of the epidemic and the geographical distribution. Overall, subtype C is responsible for half of the current infections (48%), followed by subtypes A (12%), and B (11%). A high prevalence of recombinant forms, which account for at least 21% of HIV-1 infections worldwide, is also noteworthy (Hemelaar, 2012). As seen in Latin America and the Caribbean countries, subtype B prevails in most parts of Brazil, followed by subtypes F1, C, D, and diverse recombinant forms. Southern Brazil, however, presents a distinct epidemiological pattern, with a higher prevalence of subtypes C, B, and BC recombinants (Cardoso et al., 2009; Machado et al., 2009, 2017; de Medeiros et al., 2011; Almeida et al., 2012; Graf and Pinto, 2013; Velasco-de-Castro et al., 2014; Junqueira and Almeida, 2016; Pessoa et al., 2016; Delatorre et al., 2017; Filho and Brites, 2017; Lima et al., 2017).

Through the progress of next-generation sequencing (NGS) techniques it became possible to expand the study of HIV genetic diversity, evolutionary and epidemic processes, allowing the generation of HIV complete or near full-length genomes (NFLG) and improving the characterization of intra- and interhost diversity of viral populations. Greater sensitivity and accuracy in the detection of viral recombinant forms represents one of the major improvements associated with this development, since most of the previous studies were based on partial HIV genomic sequences, mainly within the *pol* gene region due to the interest in determining drug resistance mutational patterns, resulting in underestimation of the frequency of recombinant forms (Thomson and Najera, 2005; Hemelaar et al., 2011; Marques et al., 2018). It also permits the characterization of multiple viral infections within individual hosts. Previous Brazilian studies using NGS to analyze HIV diversity were able to identify several distinct unique and circulating recombinant forms and evidenced dual infections (Pessoa et al., 2014b, 2015, 2016; Alves et al., 2017; Marques et al., 2018). In the pooled analysis depicted in this report, we pooled publically available Brazilian sequences obtained by NGS and new genetic data from HIV-positive patients in five states of Brazil with some of the highest HIV prevalence, three in the Southeast (Rio de Janeiro, São Paulo, and Minas Gerais), one in the Northeast (Pernambuco) and one in the South (Rio Grande do Sul). Upon analyzing the HIV subtypes of this large cohort, our study was able to unveil, with unprecedented accuracy, high rates of viral recombination and highlighted that novel recombinants are continually arising in the Brazilian epidemic.

## Materials and Methods

### Study Population and Sample Collection

A total of 84 convenience samples were used for generating the experimental data presented in this study. These were from HIV-1-seropositive patients recruited between February 2016 and December 2017 during the routine services conducted at Sexually Transmitted Diseases/HIV ambulatory at Hospital Federal de Ipanema (HFI) and at Hospital Universitário Clementino Fraga Filho (HU-UFRJ), both located in Rio de Janeiro, southeastern Brazil, and at Hospital Universitário Dr. Miguel Riet Corrêa Jr. (HU-FURG), located in Rio Grande, southern Brazil. Clinical and epidemiological data were obtained through a questionnaire and 10 ml of whole peripheral blood were collected. This research was approved by the Ethics Committees in Research of the Brazilian National Cancer Institute – INCA and of HFI (CAAE 52862016.9.0000.5274), HUCFF-UFRJ (CAAE 56604816.2.0000.5257), and HU-FURG (CAAE 52862016.9.3001.5324). The inclusion criteria were being 18 years or greater, being under first antiretroviral scheme and being upon virological success (undetectable HIV viral load) for the last 12 months. A fraction of this casuistic, 32 patients from HFI, has been previously described (Alves et al., 2017). We pooled these data with all Brazilian HIV-1 data comprising NFLG and partial sequences determined by NGS and publically available (Pessoa et al., 2015, 2016). All studies included in the present report (either experimentally determined herein or retrieved from the literature) used a similar methodology to amplify the HIV NFLG and sequence them in an Illumina MiSeq platform. Multiple infection analyses were also made by *de novo* assembly as described below.

### DNA Extraction and PCR of Proviral DNA

The patients’ genomic DNA containing their HIV-1 proviruses was extracted from whole blood with the Genomic DNA Extraction Kit (Real Genomics, BioAmerica, Inc.) following manufacturer’s instructions. Nested PCR performed using Platinum^TM^ Taq DNA Polymerase High Fidelity (Life Technologies) was carried out in a Veriti^®^ 96-Well Thermal Cycler (Life Technologies, Carlsbad, United States) for the amplification of HIV NFLGs. The strategies comprised the amplification of four to five fragments, from 2 to 3 kb each, spanning the whole HIV genome (Sanabani et al., 2006b; Ode et al., 2015). After visualization using GelRed (Biotium, Hayward, CA, United States) in 1% agarose gels, duplicated independent PCR-positive products were pooled directly to avoid representativeness of PCR-based errors and their interpretation as minority variants in the population. PCR products were purified with the GFX^TM^ PCR DNA and Gel Band Purification Kit (GE Healthcare, MA, United States) and their concentration was measured in a NanoDrop ND 1000 apparatus (Thermo Fisher Scientific, MA, United States). The purified products were diluted to 4 ng/μL and pooled per patient.

### Library Construction and NGS

Libraries were prepared with the Nextera XT DNA Sample Preparation kit (Illumina Inc., San Diego, United States) according to the manufacturer’s protocol, except that the starting material was diluted to 0.4 ng/μL. The library construction consists of a fragmentation step using transposon technology, followed by a PCR step where dual indexes were added to the fragments. After this process, libraries were quantified by qPCR with the KAPA library quantification kit (Kapa Biosystems, MA, United States) or by fluorometric quantitation with the Qubit dsDNA HS Assay Kit (Life Technologies, Carlsbad, EUA). Libraries were diluted, and pooled prior to sequencing in a MiSeq Illumina platform (2 × 301 cycles paired-end run) (Illumina) with 1% denatured PhiX DNA as a sequencing control.

### Data Analysis

The analysis of the obtained files was performed in Geneious v.9.1.3 using the same alignment parameters for a reference-based genome assembly described by Dudley et al. (2014). Reads were assembled using an annotated HIV-1 HXB2 reference sequence and 10 iterations to obtain each viral genome sequence. The presence of multiple infections was investigated by *de novo* assembly performed with IVA (*Iterative Virus Assembler*) with default parameters (Hunt et al., 2015). This assembler is based on seed sequences that are iteratively and conservatively extended into a contig using reads that have a perfect match. After this process, the program uses those contig as reference in a reference genome aligner to extend the initial contigs obtained (Hunt et al., 2015). The contig sequences obtained were submitted to a BLAST nucleotide analysis to discard those of human origin and confirmed with the Los Alamos National Laboratories HIV BLAST Tool^2^. All samples suggestive of multiple infections (more than one contig representing the same genomic region) were re-aligned using their respective IVA-assembled contigs as references in the Geneious program. More restrictive assembly parameters were used, such as allowing mapping of paired reads only when both mapped to the same contig and discarding reads that mapped simultaneously to more than one contig. To investigate if these contigs were derived from the same virus, sequences were submitted to hypermutation analysis using the Hypermut2.0 tool available at Los Alamos HIV Database. The contigs were considered hypermutated if the *p*-value was ≤0.05 when comparing the number of APOBEC G-to-A signature mutations with the control context. The overlapped region between the contigs obtained per sample was compared to all sequences publically available at the BLASTn Database^3^. The top 10 hits of each contig were retrieved from this database to construct phylogenetic trees and check its clustering profile.

### Phylogenetic Analysis

The consensus sequence for each sample was extracted from the reference-guided assembly described above using the 50% stringency setting and classified using maximum likelihood phylogenetic analysis performed with PhyML v.3.0 and the best model of nucleotide substitution defined with Model Generator (Keane et al., 2006; Guindon et al., 2010). To investigate HIV-1 recombination the *bootscaning* tool of Simplot v.3.5.1 was used with the following parameters: *window* = 400 pb; *steps* = 40 pb; *T/t* = 2.0; *gapstrip* = on; *replicas* = 100; *nucleotide substitution model* = F84; *method* = Maximum Likelihood (Lole et al., 1999). Phylogenetic analyses were repeated for recombinant sequences considering the *bootscanning* breakpoint analysis (data not shown). The sequences obtained in this study were submitted to the GenBank under the accession numbers MK041550-MK041589. The raw sequencing reads were deposited to the Sequence Read Archive (SRA) under the numbers SRR7993842-SRR7993872.

## Results

In this study, we included data previously published by our group from patients followed-up at HFI and all HIV-1 sequences obtained by NGS publicly available. A detailed description of the studied populations can be found in their respective articles (Pessoa et al., 2015, 2016; Alves et al., 2017). We focused on the HIV-1 subtype classification, identification of HIV recombinants and multiple infection investigation for our novel patients. As previous published, the patients from HFI were mostly males (75%) with a median age of 38 years at the time of sample collection. Regarding our new cohort, we also found a prevalence of males (67%) among the patients from HU-UFRJ with a median age of 43.5 years. Unlike the other centers, a greater number of female patients (62%) were observed among patients from HU-FURG, with a median age of 43 years. Clinical and epidemiological characteristics of the three cohorts are compiled in Table 1. Regarding antiretroviral treatment, 19 patients from HI (19/32, 59%), four patients from HU-UFRJ (4/12, 33%), and 23 patients from HU-FURG (23/40, 58%), were under the HAART scheme preconized by Brazilian Ministry of Healthy at the time of sample collection, composed of tenofovir (TDF), lamivudine (3TC), and efavirenz (EFV). All schemes used by the patients are described in Table 2.

**Table 1:** Demographic and clinical characteristics of HIV-positive patients.

Characteristic	HI (*n* = 32)	HU- UFRJ (*n* = 12)	HU-FURG (*n* = 40)
Males (%)	24 (75%)	8 (67%)	15 (38%)
Age (years)	38	43.5	43
(median; IQR_50_)	(31.5–45.25)	(41–52)	(35–49.5)
Median baseline CD4+ T-cell counts	712.5	1117^1^	780.5
(cells/mm^3^; IQR_50_)	(606.5–856)		(680.75–938.5)
Median baseline CD8+ T-cell counts	657.5	NA	848
(cells/mm^3^; IQR_50_)	(529–1047.25)		(750.5–1053)
Median time since HIV diagnosis	4.7	13.3	4.83
(years; IQR_50_)	(3.9–6.5)	(7.7–14.8)	(2.42–10.9)
Median time from HIV diagnosis to start of ARV treatment	1.2	1.7	0.88
(years; IQR_50_)	(0.6–2.8)^2^	(0.1–5.3)	(0.25–3.2)
Median treatment time	3.1	9.34	3.83
(years; IQR_50_)	(2.4–3.9)	(4.6–11.4)	(2.2–4.77)


*^1^Information available for only two patients (1201 and 1033 cells/mm^*3*^). ^*2*^Data based on 31 patients. NA, information not available. IQR_*50*_, interquartile range.*

**Table 2:** Distribution of subtypes and HAART regimen exposure across the 48 HIV-1 genome sequences analyzed.

Patient	Subtype/URF	HAART Regimen	Patient	Subtype/URF	HAART Regimen
1-HI	B	AZT+3TC+NVP	**31-HI**	**B**	TDF+3TC+EFV
**2-HI^#^**	**B**	AZT+3TC+EFV	**32-HI**	**BF**	TDF+3TC+EFV
**3-HI**	**B**	TDF+3TC+EFV	**2-HU-UFRJ**	**B**	TDF+3TC+EFV
4-HI	B	TDF+3TC+EFV	3-HU-UFRJ	B	AZT+3TC+EFV
**5-HI**	**B**	AZT+3TC+ATV	**4-HU-UFRJ**	**B**	AZT+3TC+EFV
6-HI	F1	AZT+3TC+ATV/r	**6-HU-UFRJ**	**B**	AZT+3TC+EFV
8-HI	BC	TDF+3TC+EFV	**7-HU-UFRJ**	**B**	AZT+3TC+EFV
**11-HI**	**BF**	TDF+3TC+EFV	**9-HU-UFRJ**	**F1**	AZT+3TC+AZT/r
**12-HI**	**B**	TDF+3TC+EFV	**10-HU-UFRJ**	**B**	TDF+3TC+EFV
**13-HI**	**B**	TDF+3TC+EFV	**12-HU-UFRJ**	**B**	TDF+3TC+EFV
**14-HI**	**B**	AZT+3TC+LPV/r	**1-HU-FURG**	**C**	TDF+3TC+EFV
**15-HI**	**B**	TDF+3TC+EFV	2-HU-FURG	C	TDF+3TC+EFV
**16-HI**	**B**	AZT+3TC+FPV/r	3-HU-FURG	BC	AZT+3TC+EFV
**17-HI**	**BF1**	AZT+3TC+ATV/r	**6-HU-FURG**	**C**	TDF+3TC+AZT/r
**18-HI**	**B**	TDF+3TC+EFV	**7-HU-FURG**	**C**	TDF+3TC+DRV/r
**19-HI**	**B**	AZT+3TC+EFV	**8-HU-FURG**	**C**	TDF+3TC+ATV
20-HI	B	TDF+3TC+EFV	10-HU-FURG	C	TDF+3TC+EFV
**21-HI**	**B**	TDF+EFV+FTC	12-HU-FURG	C	TDF+3TC+EFV
**22-HI**	**B**	TDF+3TC+EFV	13-HU-FURG	C	TDF+3TC+EFV
**23-HI**	**B**	TDF+3TC+EFV	14-HU-FURG	BF1	AZT+3TC+AZT/r
26-HI	B	TDF+3TC+EFV	16-HU-FURG	C	TDF+3TC+EFV
27-HI	B	TDF+3TC+EFV	17-HU-FURG	C	AZT+3TC+LPV/r
28-HI	BF	TDF+3TC+EFV	18-HU-FURG	BF1	TDF+3TC+EFV
29-HI	B	TDF+3TC+EFV	20-HU-FURG	BC	TDF+3TC+EFV


*^#^The near full-length genomes are represented in bold.*

Overall, we were successful in sequencing by NGS at least 2 of the 4/5 overlapping PCR fragments of 48 samples from our cohort included in this study (57%, 48/84). Of those, 28 samples (58%; 28/48) had the NFLG obtained. The remaining partial genome sequences had complete Gag CDS (coding sequence) for nine samples (45%, 9/20), Pol CDS for 4 (20%, 4/20), and Env CDS for 12 (60%, 12/20). Six samples from HI, 4 from HU-UFRJ, and 26 from HU-FURG (43%) failed to have more than one viral DNA fragment PCR-amplified and were excluded from further analyses. Of the 302 NGS Brazilian sequences previously available in the literature, which information was also included in this study, 247 were NFLG and 55 were partial sequences (Pessoa et al., 2015, 2016).

**FIGURE 1:**
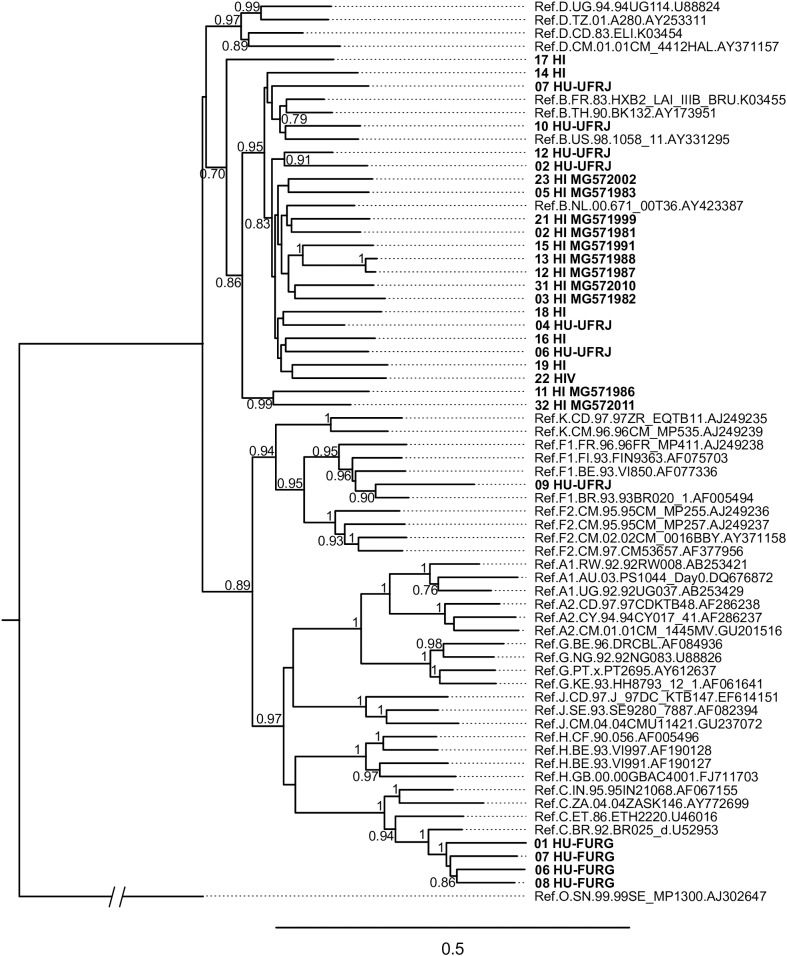
Phylogenetic analysis of HIV near full-length genomes (NFLG) from this study. The maximum likelihood analysis was performed with 1,000 bootstrap iterations. The tree contains 28 HIV-1 proviral sequences obtained from Hospital Federal de Ipanema (HFI), Hospital Universitário Clementino Fraga Filho-UFRJ (HU-UFRJ), Rio de Janeiro, and Hospital Universitário Dr. Miguel Riet Corrêa Jr. (HU-FURG), Rio Grande do Sul (represented in bold) and reference sequences of HIV-1 subtypes (named by subtype, country, year, and GenBank accession number). HI sequences determined in a previous study (Alves et al., 2017) are named with their respective GenBank accession numbers. Only bootstrap values greater than 0.7 are shown.

**FIGURE 2:**
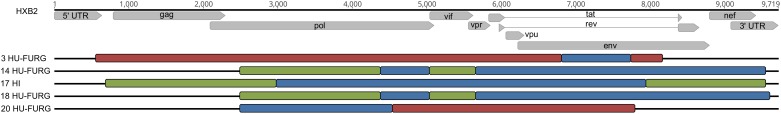
Classification of HIV-1 recombinant viruses. The recombinant patterns were defined by phylogeny and similarity analyses. Each color represents a different subtype: red for subtype B, green for subtype F1, and blue for subtype C. Samples are identified before their respective virus structure and the HXB2 reference genome sequence is at the top of the Figure for reference positioning purpose.

HIV-1 consensus sequences were subjected to phylogenetic analysis to determine their subtype/CRF classification. The NFLGs obtained by our group were mostly classified as HIV-1 subtype B (71%; 20/28), followed by subtype C (14%; 4/28), recombinant forms (11%; 3/28), and subtype F1 (4%; 1/28) (Figure 1). Table 2 describes the subtype classification of each sample included. The recombinants were classified as distinct URFs involving subtypes B and F1 based on Simplot analysis (Figure 2). Two of them, HI-11 and HI-32, were already described in our former study (Alves et al., 2017). The sequences of the 20 HIV-1 partial genomes were predominantly subtype B (7/20), C (6/20), and recombinants forms (6/20) (Table 2). A single subtype F1 sample was identified. With respect to the recombinants found in our study and submitted to Simplot analysis, two were identical URF_BF1 and a third one was a unique URF_BF1 previously described (HI-28), and three were distinct URF_BC strains (one of those also described, HI-08) (Figure 2; Alves et al., 2017).

**FIGURE 3:**
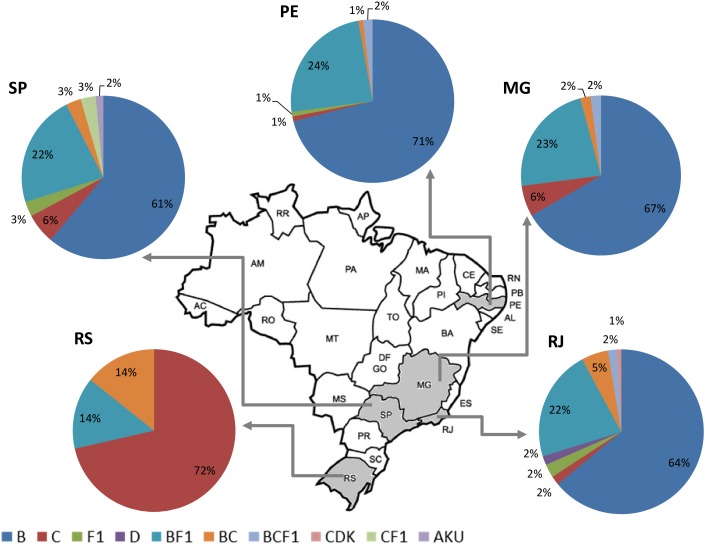
Summary of the HIV-1 subtype distribution in the five Brazilian states represented in this pooled analysis (*n* = 350). Each pie graph represents one state as depicted in the map. HIV-1 subtypes and recombinants are color-coded according to the legend at the bottom of the figure.

Altogether, the pooled analysis of all Brazilian NGS data, including NFLG (29%, 79/275) and partial sequences (29%, 22/75), showed a significant prevalence of recombinant forms (29%; 101/350). Considering the distribution of the HIV-1 subtypes and recombinant viruses in the five Brazilian states analyzed, three in the Southeast (Rio de Janeiro, São Paulo, and Minas Gerais), one in the Northeast (Pernambuco) and one in the South (Rio Grande do Sul), we could observe a highly diversified pattern of HIV-1 subtype distribution (Figure 3). A higher prevalence of recombinant forms in São Paulo and Rio de Janeiro (30%) could also be found, followed by Rio Grande do Sul (28%), and by Minas Gerais and Pernambuco (27%), although those differences were not significant (data not shown).

**FIGURE 4:**
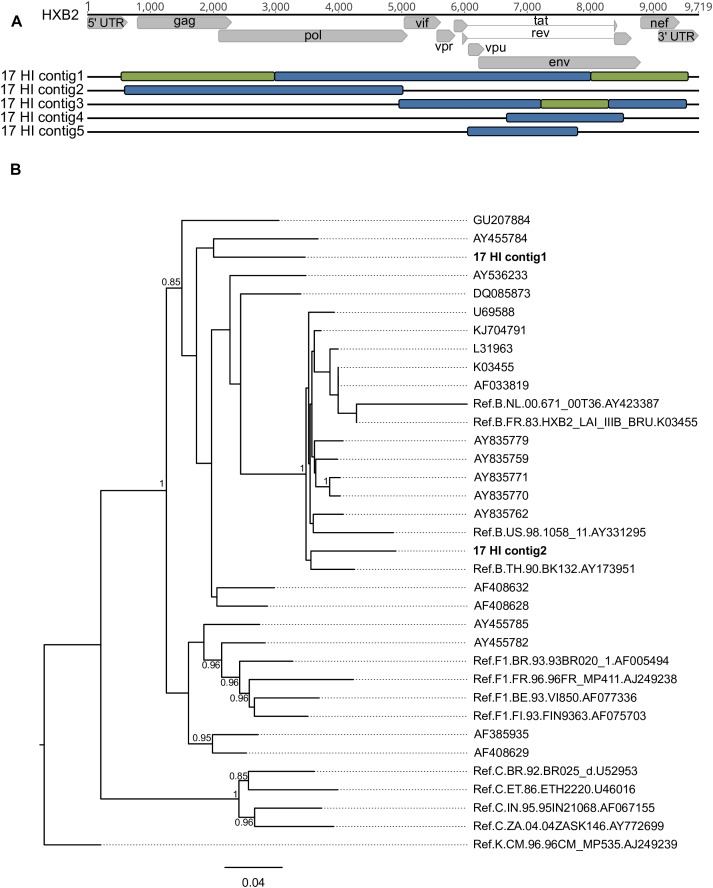
Different contigs representing HIV-1 sequences found in patient HI-17 through multiple infection investigation with their respective positions along the HXB2 reference genome and phylogenetic classification **(A)** considering the bootscaning **(B)** and maximum likelihood phylogenetic analyses. Each color represents a different subtype: red for subtype B, green for subtype F1 and blue for subtype C. Samples are identified before their respective virus structure and the HXB2 reference genome sequence is at the top of the Figure for reference positioning purpose.

*De novo* analysis generated results suggestive of multiple infections (more than one IVA-contig in the same genomic region) for four samples (HU-UFRJ-03, two contigs; HI-11, two contigs; HI-14, three contigs, and HI-17, five contigs). For three of them (HU-UFRJ-03, HI-11, and HI-14), only one of the contigs generated intact open reading frames (ORFs), while the other contigs presented truncated ORFs showing multiple stop codons, consistent with APOBEC-mediated G-to-A mutations. They were confirmed as hypermutated sequences when compared to the viable sequence from the respective patient in Hypermut (*p* < 0.05, data not shown). Patient HI-17 had several overlapping regions between the contigs, two of them at the *gag-pol* region and three at *env* (Figure 4A). Phylogenetic trees comprising the contigs, the top-ten best hits found in BLASTn searches for each contig and reference sequences were constructed for each overlapping region. Overall, these trees showed different clustering profiles between the contigs and HIV-1 subtype references, suggesting the presence of variants with different HIV-1 subtypes within this samples (Figure 4B). The contigs were then submitted to Simplot analysis to confirm the subtype classification and determine the recombination breakpoint profile. All contigs were confirmed as distinct variants. For the HI-17 patient, the longest contig had the same recombination profile observed for the consensus sequence (URF_BF1), one had a distinct recombination profile comprising B and F1 subtypes, and three were classified as subtype B (Figure 4A). Another two cases of multiple infections with distinct subtypes were described by Pessoa et al., one involving viruses of subclade F1 and subtype B and another involving a CBF1 recombinant and a non-recombinant subtype B (Pessoa et al., 2015, 2016).

## Discussion

The present study describes the HIV-1 genetic diversity and molecular epidemiology observed in Brazil using NGS-generated HIV-1 sequences, combining recently published reports and novel data from our group. In comparison to our previous published study with HIV^+^ patients recruited at HFI, we extended our analysis for three of the nine patients which data could not be obtained in the first study, and obtained NFLG sequences for another five patients (Alves et al., 2017). Concerning the integrity of the open reading frames (ORF), 4.5% (11/247) of the sequences available in the literature, and 25% (7/28) of our sequences displayed mutations and/or insertions and deletions resulting in frameshifts or premature stop codons.

Considering our cohort, a high prevalence of infection by HIV-1 subtype B viruses was found among patients from Rio de Janeiro (79%, 27/34). On the other hand, subtype C was the most prevalent subtype in Rio Grande do Sul, with a similarly high proportion (72%, 10/14), highlighting the regional differences observed in HIV-1 subtype distribution in the country. It is well documented that the overall prevalence of non-B strains, such as URF_BF1, URF_BC, and particularly subtype C and CRF31_BC in the South of Brazil, has been increasing (Santos et al., 2007; Cardoso et al., 2009; Machado et al., 2009; Almeida et al., 2012; Velasco-de-Castro et al., 2014). Similarly to some recently published data using NFLG, the recombinants identified in our cohort did not show any similarity with the CRFs already described (Sanabani et al., 2013; Alves et al., 2017). However, it is worth to mention that two partial sequences from HU-FURG show the same recombinant pattern (14 HU-FURG and 18 HU-FURG) and did not have any evidence of epidemiological linkage between them.

Through the analysis of HIV-1-positive patients in five states of Brazil with some of the highest HIV prevalence, three in the Southeast (Rio de Janeiro, São Paulo, and Minas Gerais), one in the Northeast (Pernambuco) and one in the South (Rio Grande do Sul), we could determine, with unprecedented accuracy, the prevalence of HIV-1 recombinant forms in the Brazilian epidemic and their distribution across the country. The high prevalence of recombinant strains (29%) identified by NGS is supported by the circulation of multiple subtypes and consistent with the hypothesis that novel recombinants are continuously arising in the Brazilian epidemic (Sanabani et al., 2013). Our cohort presented 19% of recombinant sequences, a lower prevalence than 28 and 40% observed in other Brazilian NGS-based studies (Pessoa et al., 2015, 2016). The higher prevalence of recombinant forms when compared to recent Brazilian Sanger sequencing-based studies (ranging from 5 to 16%) can be attributed to the smaller genomic region analyzed in the latter, mostly based only on *pol* gene, which impairs the accurate classification of recombinants (Librelotto et al., 2015; Moura et al., 2015; da Costa et al., 2016; Dos Anjos Silva et al., 2016; Delatorre et al., 2017; Filho and Brites, 2017; Lima et al., 2017). Some of these studies covering small genomic regions could not find recombinant strains, like the study conducted by Corado et al. (2017) among 73 individuals from Roraima state, northern Brazil. A study conducted by Graf et al. (2016) found a higher proportion of recombinant strains than others Sanger-based studies (21%, 66/317). However, this study used molecular data from more than one gene (HIV-1 *pol*, *env*, or both). It should also be noted that 30% of the URF_BC samples were intergenic recombinants whose recombination breakpoints were not documented within these fragments (Graf et al., 2016). The comparison of the estimated prevalence of recombinant virus between the classical Sanger-based approach and the NGS data clearly highlights underestimated rates in the former analyses, mainly associated with the smaller genomic region analyzed, which also implies in the inaccurate detection of the recombinant breakpoints and, therefore, their classification.

HIV-1 NFLG-based studies can unveil an underestimated rate of recombinant viruses in the country. Using NFLG data, several studies have described new Brazilian CRF strains designated as CRF28_BF, CRF29_BF, CRF39_BF, CRF40_BF, CRF46_BF, and CRF31_BC, CRF70_BF1, CRF71_BF1, CRF72_BF1, CRF90_BF1 (De Sa Filho et al., 2006; Sanabani et al., 2006a,b, 2010; Santos et al., 2006; Guimaraes et al., 2008; Pessoa et al., 2014a,b; Reis et al., 2017). An important study conducted by Pessôa et al. evaluated the complete genomes of HIV-1 strains by NGS previously sequenced by Alencar et al. assigned to subtype F1 and showed that 23 of the 24 samples analyzed were BF recombinants, with 4 CRF70_BF1 and 11 CRF71_BF1 novel recombinant types (Alencar et al., 2013; Pessoa et al., 2014b). The same was observed by Marques et al. (2018) where 34 of the 55 sequences analyzed were classified as recombinants. In our cohort, only one sequence was classified as subtype F1 and three as URF-BF1, which corroborates to literature and highlights the higher prevalence of recombinants forms comprising subtype F1.

Regarding molecular diversity, our pooled analysis corroborates the crescent prevalence of non-B strains in the Brazilian epidemic, confirming the phylogenetic intermixing of HIV-1 sequences. The most prominent case comprises subtype C and C-containing recombinant forms expanding from the South of Brazil to other regions (Bello et al., 2012; Graf and Pinto, 2013; Graf et al., 2015). Non-B strains represent 39% of the sequences from São Paulo, 36% from Rio de Janeiro, 33% from Minas Gerais, and 29% from Pernambuco. Subtype B was not found in Rio Grande do Sul, probably because of the small number of samples analyzed in this region.

We also investigated infection by distinct variants using a *de novo* strategy to obtain sequences from each patient that are subsequently run in one of the reference-guided approaches using this sequence as a reference (see section Data Analysis of Materials and Methods). This strategy was employed by several studies to reduce the influence of a reference genome in the assembly process while investigating multiple infections (Mangul et al., 2014; Aralaguppe et al., 2016; Alampalli et al., 2017; Baaijens et al., 2017). The prevalence of multiple infections observed in our study (2%, 1/48) was similar to the prevalence reported by Pessôa et al. (4%, 1/24 and 2%, 1/47), but both were greater than the prevalence subsequently reported (0.3%, 1/259) (Pessoa et al., 2014b, 2015, 2016, respectively). At this point, it is not possible to infer whether the distinct viral strains resulted from coinfections or the acquisition of a second variant after the establishment of the first one (superinfections).

We are aware that the pooled analysis presented here includes HIV-positive patients with different HIV clinical profiles. While our cohort is composed by patients under first-line HAART and undetectable HIV viral load for at least 12 months prior to collection date attending at sexually transmitted diseases/HIV ambulatory, the studies conducted by Pessôa et al. involved recently infected donors at four blood centers. However, it should be noted that all epidemiological HIV-1 NFLG studies based on NGS conducted in Brazil available in the literature were included in this pooled analysis. However, it should be noted that our convenience samples may have biased the analysis, especially regarding the multiple infection prevalence, which requires analysis from a larger data set.

Like previously described, the inclusion criteria used in this study were very strict and represented an important barrier to enroll a large number of patients. The difficulty at PCR-amplification of archived proviral genomes was also a limitation due to early chronic infection and undetectable HIV viral load. It is also important to mention that HU-FURG samples were incorporated in this study at a later stage. This fact, coupled with the difficulty in the PCR amplification of these samples resulted in a limited number of sequences from Rio Grande do Sul and additional studies are necessary to complement our findings. Another limitation of our study was that only the NFLG sequences (*n* = 28) were evaluated for ORF intactness, and seven of them (25%) had stop codons due to hypermutation or to frameshift deletions.

The analysis of all Brazilian HIV-1 NFLG obtained by NGS give us a more accurate evaluation of the viral diversity present in this epidemic. Through the subtype analyses conducted in this large cohort, we were able to find high rates of viral recombination, showing that larger viral genomic regions are required for reliable genetic evaluation and thus to establish effective public health policies to assure suitable HIV screening, diagnosis, monitoring and novel strategies based on viral variability. Our data highlight the great HIV genetic diversity found in Brazil and unveils a more accurate scenario of the HIV evolutionary dynamics in the region.

## Ethics Statement

This study was carried out and approved in accordance with the recommendations of the Ethics Committees in Research of the Brazilian National Cancer Institute – INCA and of HFI (CAAE 52862016.9.0000.5274), HUCFF-UFRJ (CAAE 56604816.2.0000.5257), and HU-FURG (CAAE 52862016.9.3001.5324), with written informed consent from all subjects. All subjects gave written informed consent in accordance with the Declaration of Helsinki.

## Author Contributions

BA, ES, and MS conceived the study. BA and VDH collected and processed the samples. BA, JS, IP, and OB performed all molecular biology experiments and all the bioinformatics analyses. SS contributed with previously published HIV-1 NGS sequences from Brazil. PR-P and HF contributed reagents and provided critical reading of the manuscript. BA, JS, and MS wrote the manuscript. All authors read the manuscript and agreed with its final version and submission.

## Conflict of Interest Statement

The authors declare that the research was conducted in the absence of any commercial or financial relationships that could be construed as a potential conflict of interest.

## Footnotes

1 http://www.hiv.lanl.gov/content/sequence/HIV/CRFs/CRFs.html

2 https://www.hiv.lanl.gov/content/sequence/BASIC_BLAST/basic_blast.html

3 https://blast.ncbi.nlm.nih.gov/
